# Cerebellar theta burst stimulation modulates the neural activity of interconnected parietal and motor areas

**DOI:** 10.1038/srep36191

**Published:** 2016-10-31

**Authors:** Elias Paolo Casula, Maria Concetta Pellicciari, Viviana Ponzo, Mario Stampanoni Bassi, Domenica Veniero, Carlo Caltagirone, Giacomo Koch

**Affiliations:** 1 Non Invasive Brain Stimulation Unit, Department of Behavioural and Clinical Neurology, Santa Lucia Foundation IRCCS, Rome, Italy; 2 Department of System Medicine, Tor Vergata University, Rome, Italy; 3 Stroke Unit, Tor Vergata Policlinic, Rome, Italy

## Abstract

Voluntary movement control and execution are regulated by the influence of the cerebellar output over different interconnected cortical areas, through dentato-thalamo connections. In the present study we applied transcranial magnetic stimulation (TMS) and electroencephalography (EEG) to directly assess the effects of cerebellar theta-burst stimulation (TBS) over the controlateral primary motor cortex (M1) and posterior parietal cortex (PPC) in a group of healthy volunteers. We found a TBS-dependent bidirectional modulation over TMS-evoked activity; specifically, cTBS increased whereas iTBS decreased activity between 100 and 200 ms after TMS, in a similar manner over both M1 and PPC areas. On the oscillatory domain, TBS induced specific changes over M1 natural frequencies of oscillation: TMS-evoked alpha activity was decreased by cTBS whereas beta activity was enhanced by iTBS. No effects were observed after sham stimulation. Our data provide novel evidence showing that the cerebellum exerts its control on the cortex likely by impinging on specific set of interneurons dependent on GABA-ergic activity. We show that cerebellar TBS modulates cortical excitability of distant interconnected cortical areas by acting through common temporal, spatial and frequency domains.

The cerebellum plays a fundamental role in voluntary movement control and execution. Such role is mainly exerted by modulation of the primary motor cortex (M1) through cerebello-thalamo-cortical (CTC) connections^1^. These fibers connect the dentate nucleus (DN) to M1 through the ventrolateral motor thalamus in a dysinaptic excitatory pathway^2^. Traditionally, DN output was thought to project exclusively to M1^3^,^4^. However, further evidence from animal studies using transneuronal tracers revealed that different DN regions project also to other areas connected to the thalamic relay, such as posterior parietal (PPC) and premotor cortices^2^,^4^,^5^. In addition, recent evidences showed that the cerebellum plays a key role also in high-level cognitive processes, supporting the existence of cerebellar projections to non-motor associative areas^6^.

In humans, connectivity mechanisms in the CTC pathway have been explored by means of transcranial magnetic stimulation (TMS)^7^ or transcranial electrical stimulation (tES)^8^ of the cerebellum. Oliveri and colleagues^9^ reported a reduction of motor-evoked potentials (MEPs) after 600 stimuli of low-frequency rTMS (1 Hz) over the left cerebellum. Along the same lines, Koch and colleagues^7^ used cerebellar theta-burst stimulation (TBS), a high-frequency protocol of stimulation delivered at a low intensity, able to induce changes in cortical excitability of motor^10^ and non-motor^11^ areas. In this study continuous TBS (cTBS) of the lateral cerebellum induced a reduction of short intracortical inhibition (SICI) and an increase of long intracortical inhibition (LICI) over M1, while intermittent TBS (iTBS) decreased LICI. Such changes were interpreted as a modulatory effect of cerebellar TBS on intracortical GABA circuits, in particular pointing to a possible strong modulation of GABA(B) activity as measured by LICI^7^, presumably by acting through CTC pathway.

So far, the effects of cerebellar stimulation have been tested only over M1 by means of MEPs, since TMS over non-motor areas does not produce any visible marker of excitability. Thus, possible effects on other interconnected areas, such as the PPC, cannot be revealed with this approach. Given the evidence that cerebellar output projects to different regions^4^,^5^,^6^ it is likely that neuromodulatory effects of cerebellar rTMS could be reflected also in non-motor interconnected areas. Currently, the combined use of TMS and electroencephalography (EEG) represents one of the most promising approaches in the investigation of brain dynamics^12^. Indeed, EEG is able to record the post-synaptic potentials (i.e. TMS-evoked potentials, TEPs) generated by the TMS-evoked neuronal depolarization providing accurate direct information on the neurophysiological state of the stimulated area and on its connections over all the cortex^13^. Recently, the main components of TEPs that can be detected in the first 200 ms after the probing TMS pulse have been put in relation with the activity of GABAergic interneurons, at least in M1^14^,^15^,^16^. Moreover, the analysis of TEPs in the time-frequency domain have been recently used to assess integrity of the thalamo-cortical system, which is responsible for the generation and preservation of brain’s oscillatory rhythms^17^.

Here we used TBS stimulation in a sample of healthy volunteers to induce a long lasting modulation of the lateral cerebellum. We applied TMS-EEG to measure directly changes in the neural activity of M1 and PPC, areas known to be under the influence of the cerebellar projections^4^,^5^,^6^. We hypothesized that different protocols of TBS would bi-directionally change TEPs and their related oscillatory activity by targeting specific local intracortical circuits known to be influenced by GABAergic receptors.

## Results

### TMS-evoked potentials (TEPs)

**Figure 1:**
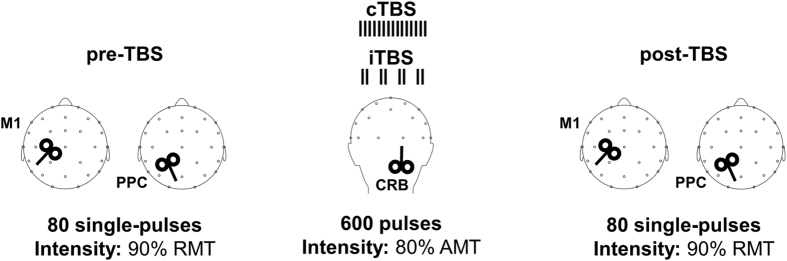
Schematic representation of the experimental procedure. Each experimental session consisted of one TBS treatment (600 pulses; 80% of AMT), intermittent (iTBS) or continuous (cTBS), and two blocks of single-pulse stimulation (80 pulses; 90% of RMT) over M1 and PPC (counterbalanced order) delivered before and 10 minutes after TBS.

**Figure 2:**
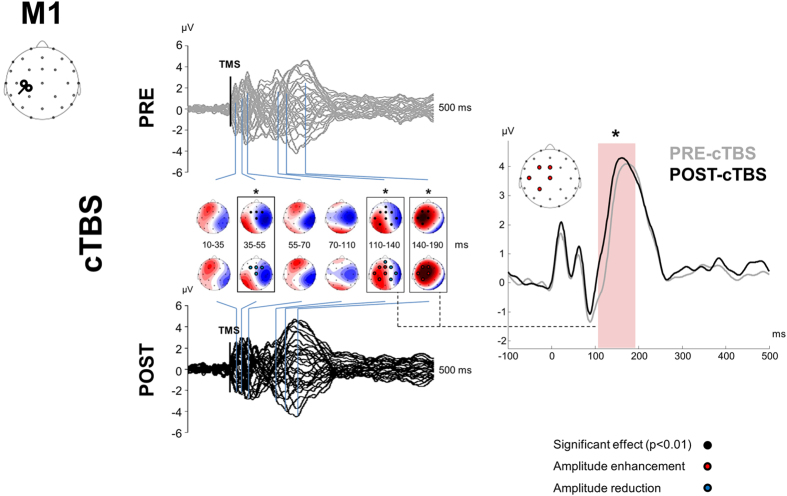
TEPs from M1 stimulation before (grey lines) and 10 minutes after (black lines) cerebellar cTBS. Left plots depict the spatiotemporal distribution of TEPs recorded from all over the scalp. Right plot depicts the TEPs recorded from a cluster of electrodes detected as significant from cluster-based permutation analysis. Black boxes indicate significant differences between pre-cTBS and post-cTBS in the time domain, black dots indicate significant differences between pre-cTBS and post-cTBS in the space domain (Monte-Carlo *p-*value < 0.01). Red and blue dots respectively indicate a significant increase or decrease of TEPs amplitude after cTBS.

**Figure 3:**
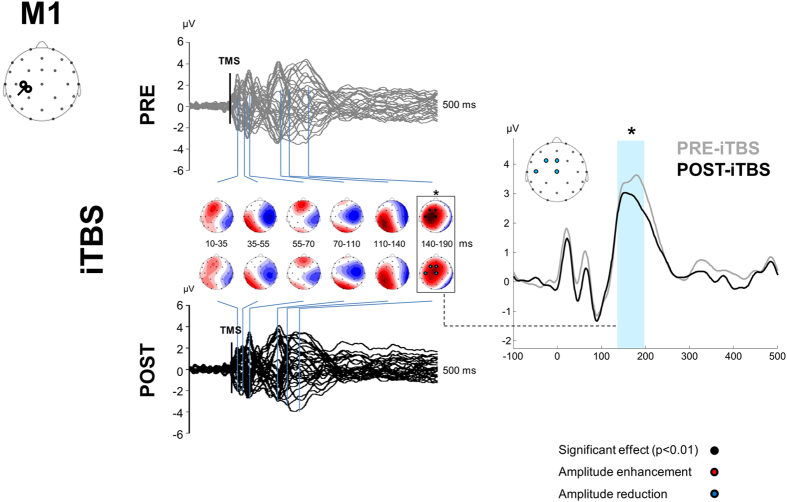
TEPs from M1 stimulation before (grey lines) and 10 minutes after (black lines) cerebellar iTBS. Left plots depict the spatiotemporal distribution of TEPs recorded from all over the scalp. Right plot depicts the TEPs recorded from a cluster of electrodes detected as significant from cluster-based permutation analysis. Black boxes indicate significant differences between pre-iTBS and post-iTBS in the time domain, black dots indicate significant differences between pre-iTBS and post-iTBS in the space domain (Monte-Carlo *p-*value < 0.01). Red and blue dots respectively indicate a significant increase or decrease of TEPs amplitude after iTBS.

**Figure 4:**
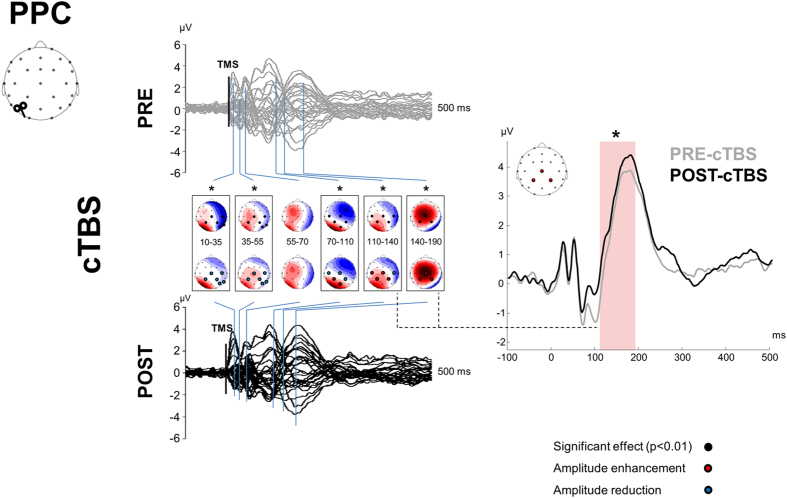
TEPs from PPC stimulation before (grey lines) and 10 minutes after (black lines) cerebellar cTBS. Left plots depict the spatiotemporal distribution of TEPs recorded from all over the scalp. Right plot depicts the TEPs recorded from a cluster of electrodes detected as significant from cluster-based permutation analysis. Black boxes indicate significant differences between pre-cTBS and post-cTBS in the time domain, black dots indicate significant differences between pre-cTBS and post-cTBS in the space domain (Monte-Carlo *p-*value < 0.01). Red and blue dots respectively indicate a significant increase or decrease of TEPs amplitude after cTBS.

**Figure 5:**
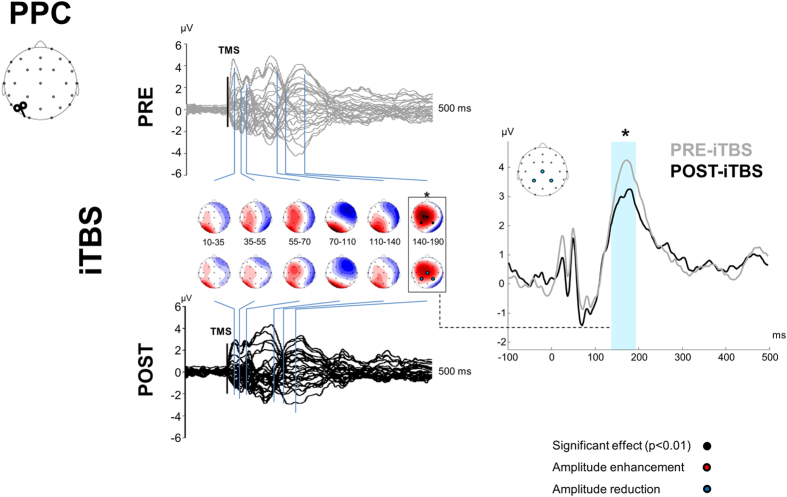
TEPs from PPC stimulation before (grey lines) and 10 minutes after (black lines) cerebellar iTBS. Left plots depict the spatiotemporal distribution of TEPs recorded from all over the scalp. Right plot depicts the TEPs recorded from a cluster of electrodes detected as significant from cluster-based permutation analysis. Black boxes indicate significant differences between pre-iTBS and post-iTBS in the time domain, black dots indicate significant differences between pre-iTBS and post-iTBS in the space domain (Monte-Carlo *p-*value < 0.01). Red and blue dots respectively indicate a significant increase or decrease of TEPs amplitude after iTBS.

Figure 1 depicts the experimental procedure. Single-pulse TMS over M1 and PPC evoked a well-known sequence of positive and negative deflections with amplitude ranging from ~−4 to ~4 μV and lasting up to ~250 ms (Figs 2, 3, 4 and 5)^12^. When stimulating M1 (Figs 2 and 3), a first peak of activity focused over fronto-central electrodes was observable at 16 ms. After 30 ms, a positivity (usually reported as P30) was observable over the site of stimulation, i.e. electrodes C3, CP1, CP5. Such activity gradually spread first over posterior regions (40 ms after TMS) and then over frontal regions (60 ms after TMS) whereas a sustained negativity, usually reported as N45, was clearly observable over the hemisphere controlateral to the stimulation. Starting from 85–90 ms a prominent negativity, usually reported as N100, was observed bilaterally over central regions. Starting from 115–120 ms from TMS, N100 spread over the right hemisphere whereas a strong and sustained positivity, usually reported as P180, was observable over the site of stimulation until around 250 ms from TMS. From the cluster-based analysis, we did not observe any significant difference between the two baselines (i.e. pre-cTBS *vs.* pre-iTBS; Monte Carlo *p*-values > 0.05). Cerebellar cTBS produced a significant modulation in TMS-evoked activity from M1 over three time windows (Fig. 2): from 35 to 55 ms after TMS we observed a reduction of amplitude over a fronto-central cluster of electrodes (Cz, FC1, FCz, FC2; Monte Carlo *p*-values < 0.01); from 110 to 190 ms we observed an increase of amplitude over a cluster of electrode surrounding the stimulated area (FCz, FC1, Cz, C3, CP1; Monte Carlo *p*-values < 0.01) whereas from 110 to 14 ms a reduction was observable in a contralateral cluster (Fz, FC2, C4, CP2; Monte Carlo *p*-values < 0.01). Cerebellar iTBS produced a significant decrease of TEPs amplitude evoked from 140 to 190 ms after TMS in a cluster of electrode surrounding M1 (FCz, FC1, Cz, C3; Monte Carlo *p*-values < 0.01) (Fig. 3). TEPs pattern evoked from PPC was similar in terms of amplitude and latency to the one evoked over M1 (Figs 4 and 5). Similarly to what occurred over M1, from 10 to 55 ms after TMS of the PPC, cerebellar cTBS produced a significant TEPs reduction over a bilateral centro-parietal cluster of electrodes (Cz, C4, CP1, CP2, P4, P6; Monte Carlo *p*-values <0.01). From 70 to 110 ms after TMS, cTBS produced a significant increase of TEPs amplitude over CP1 and Pz whereas a reduction was observable over Cz and CP2 (Monte Carlo *p*-values < 0.01). Finally, from 110 to 190 ms after TMS we observed a bidirectional TBS-dependent modulation of TEPs amplitude over a centro-parietal cluster (Cz, CP1, CP2) which was significantly enhanced/reduced by cTBS (Fig. 4) and iTBS (Fig. 5), respectively. In the control experiment no significant effects were observed after sham stimulation (Monte Carlo *p-*values > 0.05).

### Local mean field power (LMFP)

**Figure 6:**
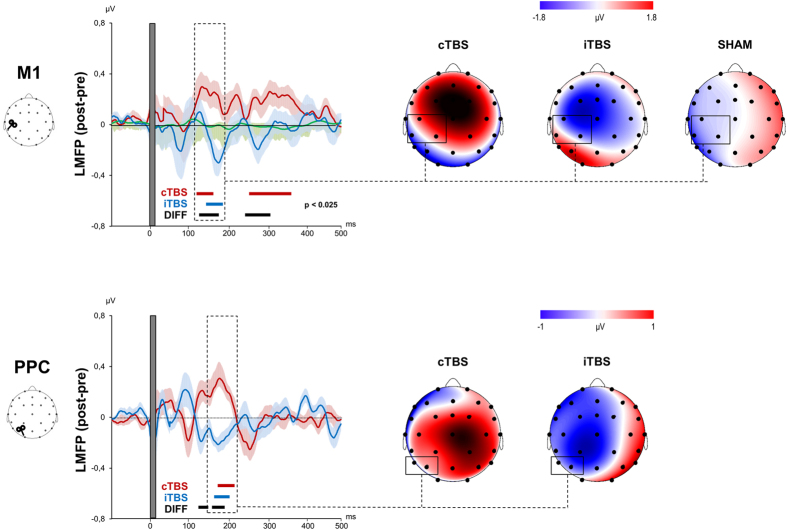
LMFP and scalp voltage maps of the post-pre cTBS (red line), iTBS (blue line) and sham (green line) differences evoked from M1 (upper panels) and PPC (below panels). Blue and red thick lines indicate significant differences (*p* < 0.025) between pre and post-cTBS, pre and post-iTBS, respectively. Black lines indicate significant differences (*p* < 0.025) between the two TBS protocols (post-pre differences).

M1-LMFP showed three main peaks of activity with maximum amplitude at ~60, ~100 and ~180 ms after TMS. Differently, PPC-LMFP showed only two distinguishable peaks of activity peaking at ~100 and ~180 ms after TMS and a generally lower amplitude compared to M1. Statistical analysis revealed a significant increase of M1-LMFP (135–179 ms; mean *p*-value = 0.025) and PPC-LMFP (182–217 ms after TMS; mean *p*-value = 0.014) after cerebellar cTBS. Cerebellar iTBS produced a significant decrease of both M1-LMFP (165–201 ms after TMS; mean *p*-value = 0.017) and PPC-LMFP (166–197 ms after TMS; mean *p*-value = 0.011) in the same time window. Figure 6 depicts the post-pre LMFP differences for M1 and PPC stimulation. LMFP evoked from M1 was oppositely modulated by cTBS and iTBS over two time windows: 139–190 ms (mean *p*-value = 0.004) and 265–320 ms (mean *p*-value = 0.006); when stimulating PPC, differences were detected at 127–145 ms (mean *p*-value = 0.009) and 165–215 ms (mean *p*-value = 0.005). No significant differences were detected before and after sham TBS nor between baselines of the three conditions (*p* > 0.05).

### TMS-related spectral perturbation (TRSP)

**Figure 7:**
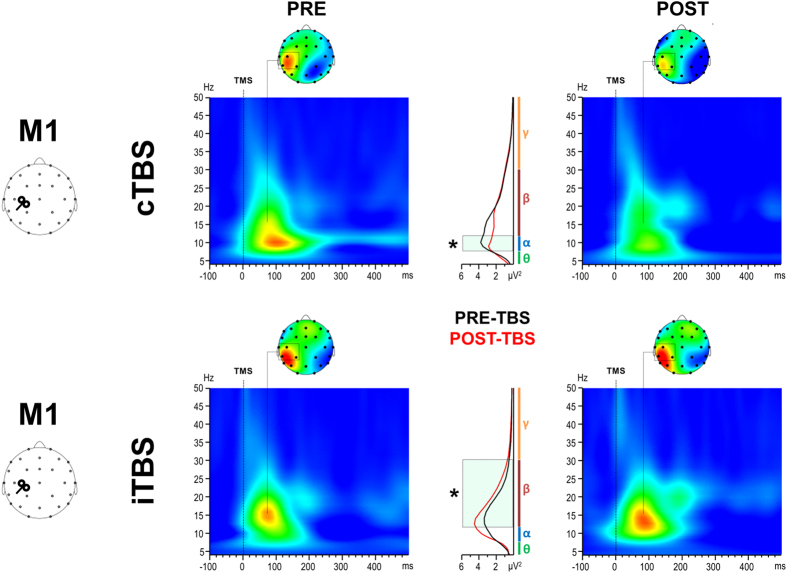
TRSP evoked from M1 before (left panels) and after (right panels) cerebellar cTBS (upper panels) and iTBS (lower panels). Middle panels depict the spectral power for each frequency band before (black line) and 10 minutes after (red line) the two TBS protocols. Black boxes indicate significant differences between pre-TBS and post-TBS in the oscillatory domain (*p* < 0.05).

**Figure 8:**
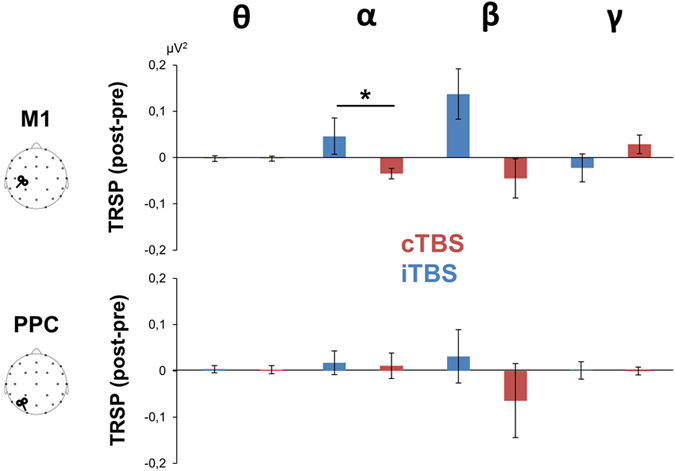
TRSP of the post-pre cTBS (red bars) and iTBS (blue bars) differences evoked from M1 (upper panel) and PPC (below panel). Asterisks indicate significant differences (*p* < 0.05).

Figure 7 depicts the TRSP plots of the TMS-induced oscillatory activity over M1 before and after TBS. Single-pulse TMS induced oscillatory activity lasting up to 150–200 ms mainly in the alpha and beta range (7–30 Hz) which appeared to be modulated in a bidirectional way by both TBS protocols. ANOVA on TRSP values revealed a significant TBS × Time × Frequency interaction [F(1.66,14.96) = 5.19; *p* = 0.024; η^2^ = 0.366]. *Post-hoc* analysis showed that alpha activity was significantly reduced by cTBS (*p* = 0.009) whereas beta activity was significantly enhanced by iTBS (*p* = 0.015). Scalp maps of TRSP showed that these effects were prominent over the M1-ROI (i.e. C3, CP1, CP5). To directly compare the two TBS protocol, an additional ANOVA was conducted on the post-pre TRSP differences (Fig. 8). This analysis confirmed the significant TBS × Frequency interaction [F(1.66,14.96) = 5.19; *p* = 0.024; η^2^ = 0.366] and showed that alpha activity was significantly modulated in a bidirectional way; specifically, cTBS resulted in an decrease whereas iTBS increased the activity (*p* = 0.017). Modulation of beta activity did not reach the significant threshold (*p* = 0.09). Single-pulse TMS over PPC induced oscillatory activity mainly in the alpha and theta range (5–9 Hz). Although some modulation were appreciable, ANOVA on TRSP values did not reach the statistical threshold (*p* > 0.05). Again, ANOVA did not reveal any difference between baselines (i.e. pre-cTBS *vs.* pre-iTBS). In the control experiment we did not observe any effect of sham TBS (*p* > 0.05).

## Discussion

We investigated the direct neurophysiological effects of cerebellar TBS over different interconnected areas of the contralateral cortex. Cerebellar TBS strongly influenced TMS-evoked activity over both M1 and PPC areas with specific characteristics over time, space and frequency. Specifically, cTBS increases whereas iTBS decreases local TMS-evoked activity between 100 and 200 ms after stimulation of M1 and PPC. Notably, no effects were observed after sham stimulation, suggesting that they were due to TBS and not to confounding effects. When investigating TEPs evoked from M1, cerebellar cTBS first decreased cortical activity over a cluster of fronto-central electrodes (35–55 ms after TMS) and then, from 110 ms after TMS, strongly increased activity over M1, affecting also the controlateral hemisphere in the opposite direction. Notably, from 140 to 190 ms after TMS, TBS effects were focused in the same cluster of electrodes surrounding M1, in which activity was enhanced/reduced by cTBS and iTBS, respectively. Interestingly, we observed the same dynamic when testing PPC cortical activity, although the effects were more focused on central and posterior electrodes of both hemispheres. The effect of iTBS were slightly later in time as compared to cTBS and more focused over the stimulated areas. Crucially, iTBS induced an opposite effect decreasing instead of increasing TMS-evoked activity at these later latencies (~140–190 ms after TMS). The analysis of LMFP confirmed the observed bidirectional TBS-dependent effects, which were detected in a similar, but shorter, time window both for M1 and PPC.

The origin of TEPs has been recently clarified by a growing body of evidence demonstrating that early TEPs components (from ~15 to ~60 ms after TMS) are likely produced or modulated by fast GABA(A)-mediated inhibitory post synaptic potentials (IPSPs), whereas later TEPs components (from ~100 to ~250 ms after TMS) are under the influence of slower GABA(B)-mediated IPSP^14^,^15^,^16^,^18^,^19^. In particular, several studies found a consistent relation between the amplitude of TEPs and the amount of GABA-ergic inhibition as assessed by either the concomitant administration of GABA-agonist^15^,^20^ or by measuring other GABA-ergic dependent MEP measures (i.e. LICI, SICI, cortical silent period)^15^,^16^,^21^,^22^. In the current study we found that cTBS increases whereas iTBS decreases TMS-evoked activity from ~100 to ~200 ms over both M1 and PPC areas. Such effect is consistent with our previous work showing that cerebellar cTBS increases while cerebellar iTBS decreases LICI^7^ a protocol known to reflect GABA(B)-ergic activity. Along the same lines, analysis of global activation revealed also a significant reduction of early (i.e. GABA(A)-ergic-mediated) TEPs, after cTBS. This effect can be due to a specific effect of cTBS-induced modulations over GABA(A)-ergic activity^7^ or as a result of a GABAergic homeostatic balance according to which an increase of GABA(B)-ergic results in a reduction of GABA(A)-ergic activity^23^. Our data provide novel evidence showing that the cerebellum likely exerts its control on the cortex by impinging on specific set of interneurons through the interaction with GABA-ergic activity.

Low-intensity cerebellar TBS may have induced different plastic changes in Purkinje cells or in local interneurons, mainly affecting the ones with lower thresholds of excitability. Considering that previous animal studies showed the existence of both LTP and LTD mechanisms in the cerebellum^24^,^25^, we recently proposed that in humans TBS could induce bidirectional long-lasting changes in CTC circuits by activating similar mechanisms of cerebellar synaptic plasticity^26^. This would result in indirect changes in the DN through a tonic facilitatory drive onto the contralateral cortex with a synaptic relay in the ventral lateral thalamus. These projections could have been tuned in different ways, increasing or decreasing the activity of specific neuronal populations within the contralateral motor and parietal cortices. Our data seem to indicate that such interaction occurred similarly in both PPC and M1 areas. This could reflect the interaction with common modular cortical circuit devoted to receive the input from the cerebellum. The cerebellum could possibly control, with similar mechanisms, different cortical areas and thereby orchestrate physiological processes requiring the involvement of several interconnected cortical areas such as learning novel complex motor skills^25^. Scalp maps of voltage distribution showed that TBS effects were prominent over the electrodes closest the stimulated areas although, when the entire scalp was considered, TBS effects were observable also bilaterally over frontal, central and posterior areas. This connectivity dynamic is compatible with previous studies showing that cerebellar output projects to several areas within the fronto-parietal network including the prefrontal, premotor and parietal areas in addition to M1^4^,^5^,^6^. However, a limitation of the current study is that the low number of EEG channels used did not allow performing a source analysis of EEG activity. Thus, conclusion in the spatial domain should be taken with caution. A recent study by Harrington and Hammond-Tooke^27^ found that 30Hz-iTBS at 90% of AMT increased N100 amplitude whereas 30 Hz-cTBS at 80% of AMT (but not at 90%) produced the opposite effect. These results are in contrast with what we observed in the present work and with previous studies using cerebellar TBS^7^. However, there are several differences in the protocol of stimulation and in the analysis that can account for this discrepancy: first, the authors delivered TBS at 30 Hz instead of the standard frequency (50 Hz); second, the authors tested TBS effects only on N100 amplitude whereas in the present study we investigated the effect over all the TEP waveform and in the oscillatory activity.

Results on the oscillatory domain showed that single-pulse TMS induced oscillatory activity lasting ~200 ms whose main frequency range depended on the stimulated area. This is in agreement with previous studies showing that each area preserved its natural frequency of oscillation when stimulated with TMS, which is its natural frequency^17^. M1 stimulation generated activity mainly in the high-alpha and low-beta frequency range (11–20 Hz) whereas PPC stimulation generated activity mainly in the alpha (13–16 Hz) and low-alpha/theta range (5–8 Hz). Our results showed that only M1 oscillatory activity was modulated by cerebellar TBS. Frequency analysis showed a TBS-dependent modulation of oscillatory activity mainly between 8 and 16 Hz which was reduced by cTBS and enhanced by iTBS. However, statistical significance were reached only for alpha rhythm after cTBS application and for beta rhythm after iTBS.

It is known that cortical oscillations originate from different brain areas interactions through the thalamus, which act as a relay between subcortical areas and the cerebral cortex, the so-called thalamo-cortical system. Two types of mechanisms are thought to mediate oscillatory rhythms: one is intrinsic, depending on the interactions between specific intrinsic currents; and one is extrinsic, produced by the interplay of excitatory and inhibitory neurons. Among these, GABAergic thalamic neurons represent the main gateway through which distinct cortical areas project to multiple thalamic nuclei. In particular, the rostral pole and the rostrolateral districts of the reticular thalamus receive afferents from motor, frontal and parietal areas^28^,^29^. In view of this, it is likely that the observed modulations of oscillatory activity reflected the TBS-induced effects on GABA(B)-ergic interneurons, occurring at thalamic or cortical (or both) level. Thus, it could be hypothesized that the cTBS-induced enhancement of GABA(B)-ergic inhibition could have reduced the power of the natural frequencies of M1, whereas the opposite effect was produced by iTBS. Notably, we did not observe any significant modulation of oscillatory activity when stimulating PPC. A number of reasons could account for this lack of effect. First, it is possible that different mechanisms regulate the interactions between PPC and the thalamus, compared to M1. For instance, it is conceivable a weaker reverberance of thalamo-cortical circuits over non-primary areas such as PPC, compared to primary areas^29^. In addition, some studies demonstrated that PPC is less responsive to TMS than M1^30^. In the present study, despite we used the same intensity for M1 and PPC stimulation, TEPs from PPC were considerately smaller than those found over M1.

The current findings may have relevant implications in cognitive studies and in clinics. It is now known that the cerebellum is involved in several high-level cognitive processes^6^. Here we demonstrated, for the first time in humans, that cerebellar output directly projects also to non-motor areas. Future studies are needed to investigate cerebellar connections to associative cortical areas during cognitive performances, in particular in perceptual processes^6^. Moreover, cerebellar non-invasive stimulation is becoming a promising approach in treating several neurological disorders^31^, possibly by inducing long-lasting changes in the interconnected cerebello-thalamo-cortical networks^26^. Recently we found that cerebellar TBS when applied daily for at least two weeks may potentially alleviate symptoms in patients with Parkinson’s disease^32^ focal dystonia^33^ and improve recovery in patients with ischemic stroke^34^.

## Conclusions

The current study provides novel physiological knowledge supporting the notion that by non-invasively stimulating the cerebellum it is possible to bi-directionally modulate specific cortical circuits that receive input from the lateral cerebellum. Hence, cerebellar TBS makes possible to change at the same time and in the same direction the activity of different cortical areas forming part of the parieto-frontal network.

## Methods

### Participants and procedure

Twenty healthy volunteers (eigth females, mean age 30.1 ± 3.5 years) were enrolled for the study after giving written informed consent. All participants were right-handed as assessed with the Edinburgh Handedness Inventory. All participants were naïve to TMS and were tested for TMS exclusion criteria^35^. The experimental procedure was approved by the Local Ethical Committee (institution: Santa Lucia Foundation) and was in accordance with the Declaration of Helsinki (Sixth revision, 2008). The twenty participant were randomly assigned to two age-matched groups, one receiving real TBS and one receiving sham TBS. Each participant of the real TBS group underwent two experimental sessions in which they received iTBS or cTBS over the right cerebellum. The two TBS protocols were delivered in two different days, at least 1 week apart, in a randomized order. Sham TBS was performed by placing the coil angled away so that no current was induced in the brain^7^. To assess the effects of TBS on cortical activity we applied 80 TMS single-pulses over M1 (in both groups) and PPC (only in the real TBS group) before and after 10 minutes from the TBS protocol (Fig. 1). The order of the stimulation of the two areas was counterbalanced across participants. During the entire session participants were seated on a comfortable armchair in front of a monitor at 80 cm of distance. They were asked to fixate a white cross (6 × 6 cm) in the middle of a black screen and to keep their right arm in a relaxed position. During TMS participants wore in-ear plugs which continuously played a white noise that reproduced the specific time-varying frequencies of the TMS click, in order to mask the click and avoid possible auditory ERP responses^36^. The intensity of the white noise was adjusted for each participant by increasing the volume (always below 90 dB) until the participant was sure that s/he could no longer hear the click^37^.

### Transcranial magnetic stimulation (TMS) and electromyography (EMG)

TBS protocol was carried out using a Magstim Rapid stimulator (Magstim Company Limited, Whitland, UK), which produced a biphasic waveform with a pulse width of ~0.1 ms, and a 70 mm figure-of-eight coil. For single-pulse stimulation TMS was delivered with Magstim 200 stimulatorand a focal 50mm figure-of-eight coil. The coil was positioned and constantly monitored by means of the Softaxic neuronavigator system (E. M. S., Bologna, Italy) coupled with a Polaris Vicra infrared camera (NDI, Waterloo, Canada), using individual T1-weighted magnetic resonance imaging (MRI) volumes as anatomical reference. For PPC stimulation coil positioning was established basing on anatomical MNI coordinates (mean normalized MNI coordinates were as follows: 46, −67, 42) and orientated 15° from the midline, so that current was induced in a posterior-anterior direction^38^. For M1 stimulation the position of the coil was defined as the site in which TMS evoked the largest MEPs in the relaxed first dorsal interosseous (FDI) muscle of the right hand with an orientation at about 45° angle away from the midline. Single-pulse stimulation intensity was determined relative to the resting motor threshold (RMT), defined as the lowest TMS intensity which evoked at least five out of ten MEPs with an amplitude >50 μV peak-to-peak in the contralateral FDI at rest^39^. TBS stimulation intensity was determined relative to the active motor threshold (AMT), defined as the lowest intensity which evoked at least five out of ten MEPs with an amplitude > 200 μV peak-to-peak in the contralateral FDI during 10% of maximum contraction^40^. The cTBS protocol consisted of three-pulse bursts at 50 Hz repeated every 200 ms for 40 s, in the iTBS protocol a 2 s train of TBS is was repeated 20 times, every 10 s for a total of 190 s^10^. Both TBS protocols consisted of 600 pulses and were delivered at 80% of AMT over the lateral right cerebellum (1 cm inferior and 3 cm left to the inion)^7^,^41^. The coil was positioned tangentially to the scalp, with the handle pointing superiorly^9^. Single-pulse TMS was delivered every 4–5 s at 90% of RMT. Surface EMG was acquired from the right FDI muscle via Ag/AgCl electrodes in a belly-tendon montage using a Digitimer D360 Amplifier (Digitimer Ltd, Welwyn Garden City, UK); raw signals were sampled at 5000 Hz and band-pass filtered at 30–2000 Hz. EMG signal was on-line monitored by SIGNAL software (Cambridge Electronic Devices, Cambridge UK).

### Electroencephalographic recordings (EEG)

EEG was recorded using a TMS-compatible DC amplifier (BrainAmp 32MR plus, BrainProducts GmbH, Munich, Germany). The amplifier was optically connected to a PC with software BrainVision Recorder through which EEG was on-line monitored, and to a 32 channels EEG cap (EasyCap Inc., Herrsching, Germany). EEG was continuously recorded from 32 TMS-compatible Ag/AgCl pellet electrodes mounted on the cap according to the 10–20 international system including: Fp1, Fp2, F7, F3, Fz, F4, F8, FC5, FC1, FCz, FC2, FC6, TP9, T7, C3, Cz, C4, T8, TP10, CP5, CP1, CP2, CP6, P7, P3, Pz, P4, P8, O1, O2, Iz. Recordings were on-line referenced to the tip of the nose; the ground electrode was placed on AFz. Two additional electrodes were used to record electrooculography (EOG) in order to monitor participant eye movements and subsequently reject ocular artifacts. Skin impedance was kept below 5 kΩ. EEG signal was bandpass filtered at 0.1–1000 Hz and the sampling frequency was 5000 Hz.

### EEG pre-processing

Off-line analysis was performed with BrainVision Analyzer2 (BrainProducts GmbH, Munich, Germany), Fieldrip toolbox^42^ and EEGLAB 13.4.4^43^, running in a MATLAB environment (Version 7.9.0, MathWorks Inc., Natick, USA). Firstly, we removed the TMS artifact by applying a cubic interpolation from 1 ms before to 10 ms after the TMS pulse. The continuous EEG signal was average re-referenced and then band-pass filtered between 1 and 80 Hz (Butterworth zero phase filters). A 50 Hz notch filter was applied to reduce noise from electrical sources. The identification of artifacts (e.g. eye blinks, muscle activity, residual TMS artifacts) was made using independent component analysis (INFOMAX ICA) applied to the continuous EEG signal. Identified components were then visually inspected in terms of scalp distribution, frequency, timing and amplitude, and then removed. Signal was then segmented into epochs starting 1 s before the TMS pulse and ending 1 s after it. Afterwards, all the epochs were visually inspected and those with excessively noisy EEG were excluded from the analysis (resulting less than 3% for each participant).

### TMS-evoked potentials (TEPs) analysis

To assess the global cortical activation induced by TMS over M1 and PPC, i.e. over all the scalp, we used multiple dependent t-tests comparing TEPs waveform at each electrode within six time windows of interest (TOI), established on TEPs literature: 10–35 ms (P30 TOI), 35–55 ms (N45 TOI), 55–70 ms (P60 TOI), 70–130 ms (N100 TOI), 130–250 ms (P180 TOI), 250–360 ms (N280 TOI)^11^,^12^,^13^,^14^,^15^. A non-parametric, cluster-based permutation statistics was conducted to correct for multiple comparisons^44^. This method performs a non-parametric statistical test by calculating Monte Carlo estimate of the significance probabilities from two surrogate distributions constructed by randomly permuting the two original conditions data for 3000 times. The clusters for permutation analysis were defined as the two (or more) neighboring electrodes in which the t-value at a given time point exceeded a threshold of *p* < 0.025^44^.

### Local mean field power (LMFP) analysis

To assess the local cortical activation induced by TMS over M1 and PPC we performed a local mean field power analysis (LMFP)^45^,^46^ from 100 ms before to 500 ms after TMS. We defined two regions of interest (ROI) including the electrodes closest to the stimulated areas basing on the scalp voltage distribution maps, such electrodes were: C3, CP1, CP5 for M1; P3 and P5 for PPC. LMFP was computed as:


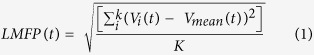


where *t* is time, *K* the number of channels, *V*_*i*_ the voltage in channel *i* averaged across participants and *V*_*mean*_ is the mean of the voltage in the channels considered for the analysis. Then, we performed a non-parametric analysis in which multiple t-tests, corrected with false discovery rate method, were computed at each time point of the LMFP waveform within the considered time window (−100 +500 ms from the TMS pulse). This analysis compared two surrogate LMFP data distributions constructed by randomly permuting the original distribution for 3000 times. Time points were considered significant when at least 10 successive t-test reached the significance threshold (*p* < 0.025)^47^.

### TMS-related spectral perturbation (TRSP) analysis

To evaluate changes in the oscillatory domain we conducted a time-frequency analysis in epochs starting 1 s before to 1 s after the TMS pulse in a frequency range starting from 4 to 50 Hz. We first performed a time-frequency decomposition for each epoch based on a complex Morlet wavelet (frequency steps = 23; cycles = *3.5*), than we computed TMS-related spectral perturbation (TRSP) as:


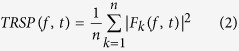


where, for *n* trials, the spectral estimate *F* was computed at trial *k*, at frequency *f* and time *t*^48^,^49^. TRSPs were baseline-normalized by subtracting the mean baseline power spectrum from each spectral estimate. Then, we extracted and averaged the TRSPs values in M1 and PPC ROIs from 10 to 300 ms after TMS in the *θ* (4–6 Hz); *α* (7–13 Hz); β (14–30 Hz) and *γ* (31–50 Hz) frequency bands for each condition and we compared them by means of a repeated-measures ANOVA with factors “TBS” (iTBS, cTBS); “frequency” (*θ, α*, β, *γ*) and “time” (pre, post). Sphericity of data was tested with Mauchly’s test; when sphericity was violated (i.e. Mauchly’s test < 0.05) the Greenhouse-Geisser correction was used. Pairwise comparisons were correct by the Bonferroni method.

## Additional Information

**How to cite this article**: Casula, E. P. *et al*. Cerebellar theta burst stimulation modulates the neural activity of interconnected parietal and motor areas. *Sci. Rep.* **6**, 36191; doi: 10.1038/srep36191 (2016).

**Publisher’s note**: Springer Nature remains neutral with regard to jurisdictional claims in published maps and institutional affiliations.
